# Comparison of buckwheat, red clover, and purple tansy as potential surrogate plants for use in semi-field pesticide risk assessments with *Bombus impatiens*

**DOI:** 10.7717/peerj.2228

**Published:** 2016-07-14

**Authors:** Angela E. Gradish, G. Christopher Cutler, Andrew J. Frewin, Cynthia D. Scott-Dupree

**Affiliations:** 1 School of Environmental Sciences, University of Guelph, Guelph, Ontario, Canada; 2 Department of Environmental Sciences, Dalhousie University, Truro, Nova Scotia, Canada

**Keywords:** Semi-field, Colony development, Bumble bee, Pesticide risk assessment, Foraging, Method development

## Abstract

**Background.** Bumble bees (*Bombus* spp.) are important wild and managed pollinators. There is increased interest in incorporating data on bumble bees into risk assessments for pesticides, but standardized methods for assessing hazards of pesticides in semi-field and field settings have not yet been established for bumble bees. During semi-field studies, colonies are caged with pesticide-treated flowering surrogate plants, which must be attractive to foragers to ensure colony exposure to the test compound, and must produce an ample nectar and pollen to sustain colonies during testing. However, it is not known which plant(s) are suitable for use in semi-field studies with bumble bees.

**Materials and Methods.** We compared *B. impatiens* foraging activity and colony development on small plots of flowering buckwheat (*Fagopyrum esculentum*, var. common), red clover (*Trifolium pratense*), and purple tansy (*Phacelia tanacetifolia*) under semi-field conditions to assess their suitability as surrogate plants for pesticide risk assessment studies with bumble bees. We also compared the growth characteristics and input requirements of each plant type.

**Results.** All three plant types generally established and grew well. Red clover and purple tansy experienced significant weed pressure and/or insect pest damage. In contrast, pest pressure was extremely low in buckwheat. Overall, *B. impatiens* foraging activity was significantly greater on buckwheat plots than red clover or purple tansy, but plant type had no effect on number of individuals produced per colony or colony weight.

**Discussion.** Because of the consistently high foraging activity and successful colony development observed on buckwheat plots, combined with its favourable growth characteristics and low maintenance requirements, we recommend buckwheat as a surrogate plant for use in semi-field pesticide toxicity assessments with *B. impatiens*.

## Introduction

Bumble bees (*Bombus* spp.) are important wild and managed pollinators of many natural and cultivated plants. There is concern and evidence of bumble bee declines in North America and Europe (Goulson, Lye & Darvill, 2008; Williams & Osborne, 2009; Brittain & Potts, 2011; Cameron et al., 2011; Colla et al., 2012; Vanbergen et al., 2013), of which there are many potential causes, including habitat loss and fragmentation (Goulson, Lye & Darvill, 2008; Williams & Osborne, 2009), climate change (Williams & Osborne, 2009; Kerr et al., 2015), population genetic factors (Cameron et al., 2011), pathogens and parasites (Goulson, Lye & Darvill, 2008; Williams & Osborne, 2009; Cameron et al., 2011), and pesticides (Goulson, Lye & Darvill, 2008; Williams & Osborne, 2009; Brittain & Potts, 2011). Of these possible contributors, pesticides by far have received the most attention from the public and scientific community. Adult bumble bees may be at risk of pesticide exposure through bodily contact with sprays or residues or consumption of contaminated pollen and nectar when foraging from treated plants, whereas developing brood may be exposed if provided contaminated pollen and nectar collected by foraging adults. Depending on the pesticide, dose, and exposure route, pesticide exposure may result in bumble bee mortality and/or various sub-lethal effects on reproduction, behaviour, and development (Gradish et al., 2010; Gradish et al., 2012; Whitehorn et al., 2012; Larson, Redmond & Potter, 2013; Smagghe et al., 2013; Gill & Raine, 2014). Toxicity data on honey bees are required for pesticide registration, and these data historically have been used to estimate risks to all other bees. However, because of their pronounced differences in physiology, life history traits, and behaviour, honey bees and bumble bees can differ in their susceptibility to pesticides (Thompson & Hunt, 1999; Devillers et al., 2003; Scott-Dupree, Conroy & Harris, 2009). Given this, and their importance as pollinators, there is increasing interest in incorporating data on bumble bees into risk assessments for pesticide registration (Vaughan et al., 2014).

Regulatory pesticide risk assessment in North America and Europe follows a tiered approach (OECD, 2010; Lee-Steere & Steeger, 2014). Initial Tier I laboratory studies function as a screen to identify pesticides that may pose a risk to bees under normal use conditions (Lee-Steere & Steeger, 2014). Pesticides that demonstrate the potential for hazard in Tier I studies are then assessed further via Tier II (semi-field) and/or Tier III (field) studies. Standardized risk assessment protocols exist for honey bees (OECD, 1998a; OECD, 1998b; OECD, 2007; OECD, 2010; EPPO, 2010), but semi-field and field methods have yet to be established for bumble bees (Cabrera et al., 2016). Preliminary semi-field and field risk assessment protocols and critical assessment endpoints for bumble bees recently have been proposed (Cabrera et al., 2016), and these methods now must be validated and refined.

Fundamental to the development of a semi-field pesticide risk assessment method for bumble bees is the identification of a suitable surrogate test plant(s). In semi-field studies, colonies are caged to pesticide-treated flowering plants in the field to determine if the pesticide has effects at the colony level (Cabrera et al., 2016). The surrogate plant used must be highly attractive to foragers to ensure colony exposure to the test compound. Additionally, the plants must produce an adequate supply of quality nectar and pollen to sustain the bees and promote optimal colony development during the test period. However, we currently lack data on the development and behaviour of bumble bees on different plants potentially used in semi-field pesticide risk assessment studies (Cabrera et al., 2016).

Here we evaluated the suitability of buckwheat (*Fagopyrum esculentum*, var. common), red clover (*Trifolium pratense*), and purple tansy (*Phacelia tanacetifolia*) as potential surrogate plants for semi-field pesticide studies with the common eastern bumble bee (*Bombus impatiens* Cresson). We focussed on these plants because they are attractive to bumble bees (Williams, 1997; Carreck & Williams, 2002; Pontin et al., 2006; Jacquemart, Gillet & Cawoy, 2007; Colla & Dumesh, 2010; Bartomeus et al., 2014), and purple tansy has previously been used in semi-field studies with bees (Cabrera et al., 2016). *Bombus impatiens* is abundant throughout eastern Canada and United States, and currently is the only bumble bee available commercially for crop pollination in North America. These attributes make it a useful surrogate species for use in bumble bee pesticide toxicity studies in North America (Cabrera et al., 2016). We compared *B. impatiens* foraging activity and colony development on small flowering plots of each plant type to determine if the plants varied in their attractiveness to foragers and/or their ability to sustain whole colonies under semi-field conditions. We also assessed the growth characteristics and input requirements of each plant type to compare the logistics and expense of their use for experimental purposes.

## Materials and Methods

### Plant establishment and maintenance

The study was conducted in a 6 ha agricultural field (soil type: sand and sandy-loam) approximately 8 km south of Tillsonburg, ON, Canada (42°48′30.62″N, 80°44′02.34″W) in 2015. The field is bordered primarily by unmanaged woodlot or residential property, with the exception of an agricultural field located across the highway to the northwest. All seeds were purchased from ProRich Seeds Inc. (Mount Elgin, ON, Canada). Between May (red clover) and June (purple tansy and buckwheat), approximately 2 ha of each plant was broadcast seeded (Sylvite Agri-Services, Springfield, ON, Canada; refer to Table S1, for specific seeding dates and details). Each plant type was seeded at the highest rate recommended for sandy soil (Table S1). Planting guidelines for all three plant types recommend that the seed be buried; however, heavy rain for 4 days immediately following seeding prevented tillage of buckwheat and purple tansy. When plants were between 2nd and 4th leaf stage, 10 plots (3.5 m^2^) were flagged. Plots were established at least 2 m apart in areas of the field where plants were evenly distributed, of similar density, and visibly healthy (i.e., not discoloured, malformed, or stunted). Plots were scouted twice weekly for the duration of the study and hand-weeded as necessary.

### Experiment set-up and data collection

For each plant type, *B. impatiens* colonies (Biobest Biological Systems Ltd., Leamington, ON), each containing a queen and approximately 20 workers, were placed in the field once the plants had reached approximately 10% bloom by visual estimate (Table S1). On the day of colony delivery, a wooden stand consisting of a plywood platform (30 × 35 cm) attached to a 5 cm^2^ stake was placed in the centre of each plot. Stands were assembled so that the platform sat approximately 10 cm above the top of the plant canopy in each tent. A screened enclosure (3.35 × 3.35 × 2.29 m, Instant Screen House^®^; Coleman Canada Inc., Brampton, ON, Canada) was then placed over each plot. Upon arrival in the field, colonies were visually inspected and weighed. Colonies containing too few workers or an abundance of ejected larvae were not included in the study. One colony was then placed on the stand in each plot.

Colonies remained in test cages for 16 days, which coincided with the shortest predicted flowering period of the three plant types (red clover, 2–3 weeks) and the proposed exposure period for semi-field pesticide risk assessments with bumble bees (Cabrera et al., 2016). Beginning the day after colonies were received, worker foraging activity on each plot was assessed every Monday, Wednesday, and Friday as follows: an observer entered the enclosure and, after 2 min to allow the bees to acclimate to the observer’s presence, made a single count of the number of workers actively foraging from flowers. The observer then recorded the number of workers entering or exiting the colony for 10 min. Temperature and relative humidity (RH) inside the enclosure was recorded on each observation period. These assessments were then repeated 1–2 h later. Therefore, there were 140 total bouts of foraging activity assessments for each plant type (10 plots × 7 observation days × 2 assessments per day). Multiple people made foraging activity observations, and thus plot assignment was randomized among observers for each assessment.

After the 16 day field period, colonies were placed in a growth cabinet at the University of Guelph and maintained in the dark at 25 °C, 20–30% RH. Each colony received 5 g of honey bee-collected pollen (Creekside Farm, Beeton, ON, Canada) three times per week and Biogluc^®^ nectar substitute (Biobest Biological Systems Ltd., Leamington, ON, Canada) *ad lib*. Colonies were monitored daily for queen emergence. Each colony was placed in a –20 °C freezer two weeks after the first emergence of a queen, or if a colony did not contain newly emerged queens or queen pupae eight weeks after removal from the field, it was frozen. All colonies were subsequently dissected to count the number of individual eggs, larvae, pupae (queen and worker/male pupae were differentiated), and adults (queens, workers, and males). Individuals were distinguished as being alive or dead when colonies were frozen: dead immature stages were brown or black, while dead adults were piled in the corners of the colony box and/or in visibly poor condition (e.g., wings splayed and tattered, lying dorsal side up, matted hair). Adult workers, males, and new queens were weighed. All new queens in each colony were weighed, but if more than 20 workers and/or males were present in a colony, 20 randomly selected individuals from each caste were weighed.

For the duration of the study, colonies (the inner plastic nest box containing the adults and brood) were weighed every Monday and Friday immediately following foraging assessments (field) or colony observations (lab).

### Data analyses

A foundress queen from a colony on a red clover plot died during the field period, and therefore this colony was excluded from all analyses (*n* = 9). Subsequently, two more queens from red clover colonies died during the lab portion of the study; these two colonies were excluded from analyses of colony development (i.e., dissection counts, adult weights, and colony weight; *n* = 7). All colonies from buckwheat and purple tansy plots were included in all analyses (*n* = 10 for each plant type).

All analyses were performed at a significance level of *α* = 0.05. A linear mixed model was used to analyze the number of active foragers on flowers per assessment and number of workers entering or exiting the colony per 10 min assessment period using the ‘nlme’ package (Pinheiro et al., 2015) in R (R Core Team, 2015). Observations were treated as a repeated measure within colonies. Variance was partitioned into the fixed effects treatment (plant type), temperature, colony weight on day of foraging observation (for foraging observations collected on Wednesdays, the mean colony weight from Monday and Friday of the same week was used), and the interaction of treatment and temperature; and the random effect colony. AIC criterion was used to determine the best-fit model, and means were compared using Tukey’s tests. The ‘effect’ package (Fox, 2003) in R was used to visualize significant interactions of the model.

Data on number of eggs, larvae, pupae and adults per colony failed to meet the assumptions of a parametric test and therefore were analyzed using Kruskal–Wallis tests in R (R Core Team, 2015). If a Kruskal–Wallis test was significant, a Wilcoxon rank sum test was performed to determine differences between means.

Data on adult worker, male, and queen weight were analyzed with a linear mixed model as before, but with the fixed effect treatment and the random effect colony. Means were compared using Tukey’s tests.

Colony weight data from the field and lab portions of the study were analysed separately using a linear mixed model as described above with observations treated as repeated measures within colonies. Variance was partitioned into the fixed effects treatment, observation day, colony starting weight, and the interactions treatment × observation day and treatment × colony starting weight; and the random effect colony. Means were compared using Tukey’s tests.

## Results

### Plant establishment, growth characteristics, and maintenance

Overall, all plant types germinated well. While purple tansy established best in areas of the field dominated by sandy-loam soil, buckwheat and red clover grew well in both sand and sandy-loam soil. Buckwheat and purple tansy began flowering 29 and 39 days after seeding, respectively, while red clover grew more slowly, requiring 66 days to flower (Table S1). Buckwheat, red clover, and purple tansy flowered for approximately 40, 18, and 30 days, respectively.

Weed pressure in red clover plots was consistently high, and thus they required weeding once or twice per week from 1st leaf stage until flower. In contrast, buckwheat and purple tansy plots were weeded only once at 3rd leaf stage; after this, their quick, dense growth supressed all weeds. Japanese beetles (*Popillia japonica* Newman) were present in the red clover in moderate to high numbers. Adults fed primarily on open flowers, and had to continually be removed by hand from the enclosures. Low numbers of Japanese beetles also were present in purple tansy, but they did not appear to cause damage to plants. However, tarnished plant bug (*Lygus lineolaris* (Palisot de Beauvois)) caused minor to moderate feeding damage to some purple tansy buds and flowers. Despite being situated between the red clover and purple tansy, neither Japanese beetle, tarnished plant bug, nor any other insect pest were present in the buckwheat.

With the exception of higher labour costs associated with weeding for red clover, the labour and input costs for all three plant types were very similar. However, purple tansy seed cost approximately seven times more than buckwheat and red clover seed (Table S1).

On Aug. 2, high winds associated with a severe thunderstorm caused lodging of the red clover plants inside and outside the enclosures. The plants were not uprooted or damaged and they continued to flower, but they remained flattened for the duration of the study. Buckwheat and purple tansy plants were not affected by the storm.

### *Bombus impatiens* foraging activity

**Figure 1:**
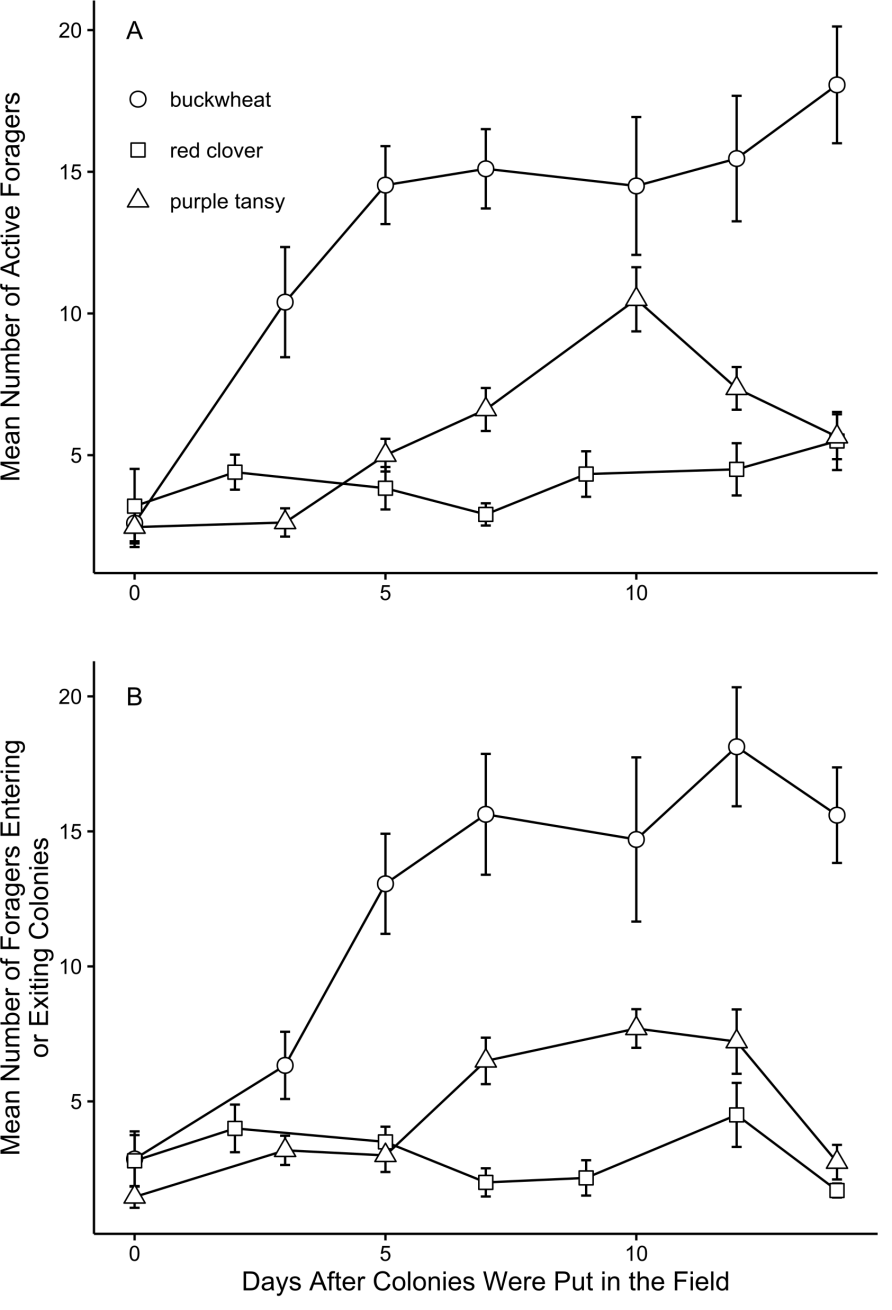
*Bombus impatiens* foraging activity over time by plant type. Mean (±SE) number of *Bombus impatiens* workers (A) actively foraging and (B) entering or exiting colonies on plots of flowering buckwheat (*n* = 10), red clover (*n* = 9), or purple tansy (*n* = 10) per assessment period on each observation day (*n* = 7).

Among the three plant types, we observed different patterns in foraging activity over time. On buckwheat plots, both the number of active foragers (Fig. 1A) and number of foragers entering or exiting colonies (Fig. 1B) increased over time. In contrast, foraging activity on purple tansy increased until observation day 5 and then began decreasing (Figs. 1A and 1B). After a small initial increase, there were only minor, intermittent changes in foraging activity on red clover plots (Figs. 1A and 1B).

**Figure 2:**
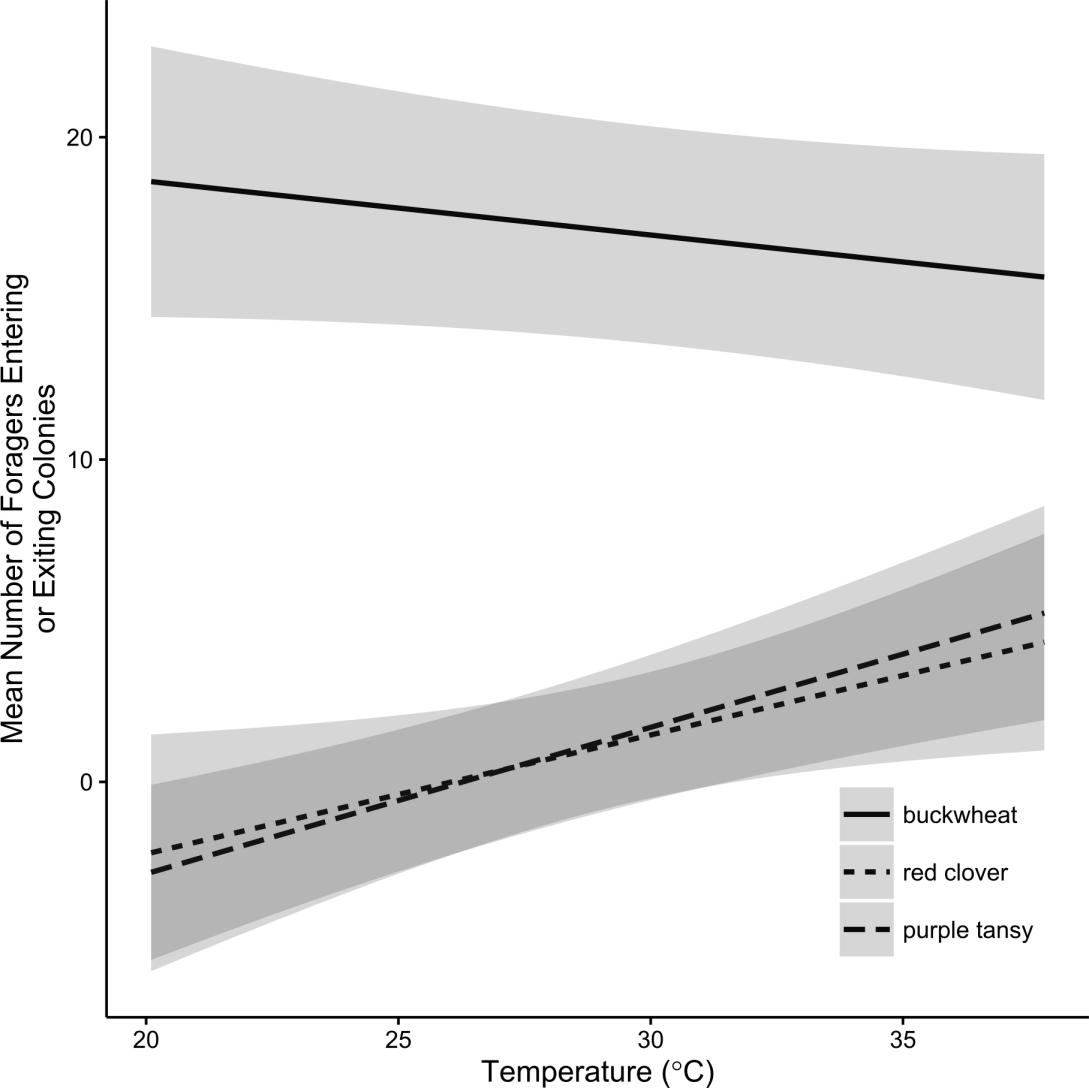
Effect display of the relationship between temperature and *Bombus impatiens* foraging activity. Effect display of the relationship between temperature (° C) and the mean number of *Bombus impatiens* workers entering or exiting colonies on small plots of flowering buckwheat (*n* = 10), red clover (*n* = 9), or purple tansy (*n* = 10) plots per assessment period over 7 observation days. Shaded areas correspond to 95% confidence intervals.

Plant type, colony weight, and temperature had a significant effect on the number of active foragers (plant type: *F* = 15.6; *df* = 2, 26; *P* < 0.0001; colony weight: *F* = 17.9; *df* = 1, 264; *P* < 0.0001) temperature: *F* = 48.2; *df* = 1, 264; *P* < 0.0001) and number of workers entering and exiting colonies (plant type: *F* = 13.2; *df* = 2, 26; *P* < 0.0001; colony weight: *F* = 24.3; *df* = 1, 264; *P* < 0.0001); temperature: *F* = 5.63; *df* = 2, 264; *P* = 0.0184). Overall, foraging activity increased with increasing colony weight and temperature. There also was a significant interaction between plant type and temperature for the number of foragers entering or exiting colonies (*F* = 6.92; *df* = 2, 265; *P* = 0.0012). While the number of foragers entering and exiting colonies was largely unaffected by temperature in buckwheat plots, it was positively associated with temperature in red clover and purple tansy plots (Fig. 2).

**Figure 3:**
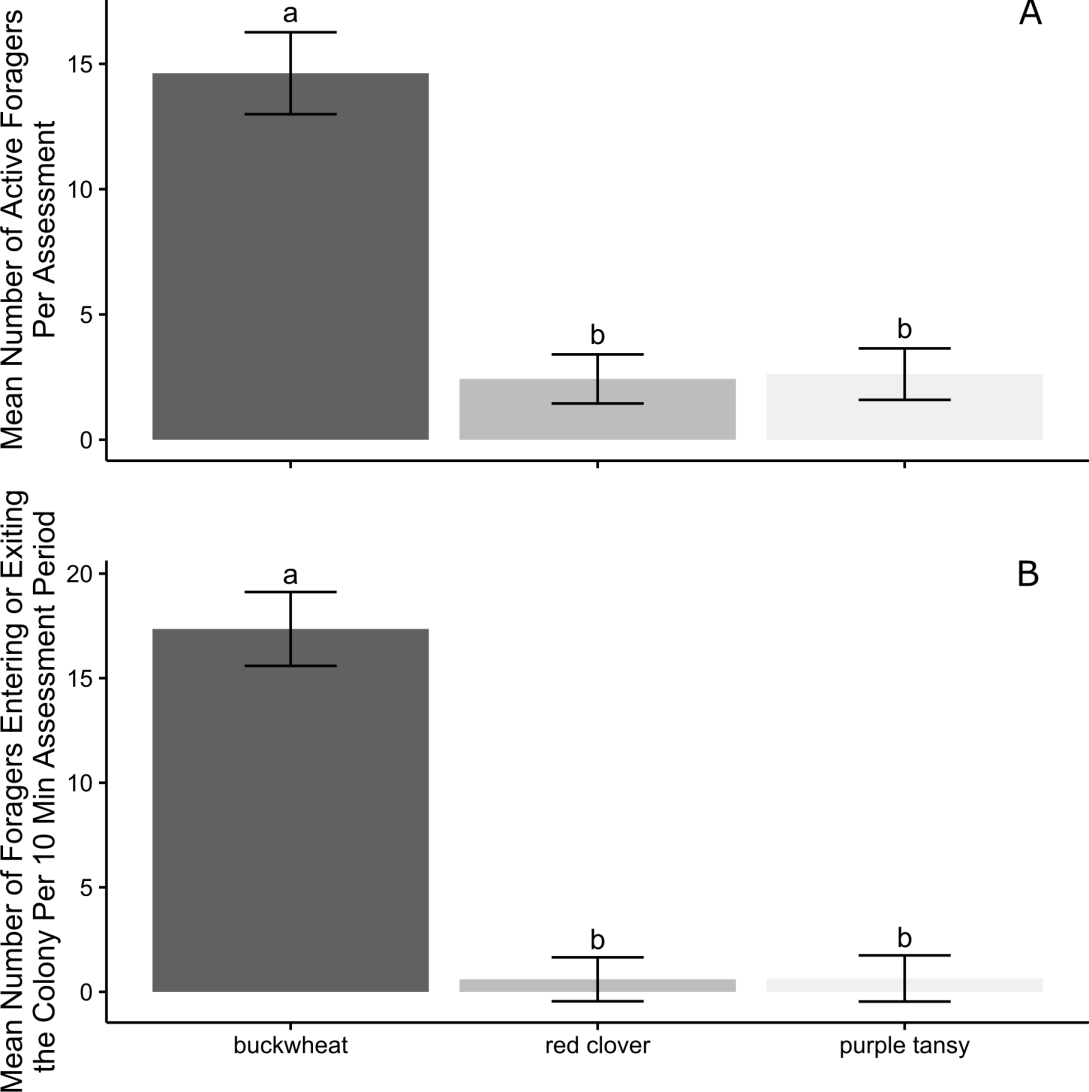
*Bombus impatiens* foraging activity by plant type. Mean (± SE) number of *Bombus impatiens* workers (A) actively foraging and (B) entering or exiting colonies on plots of flowering buckwheat (*n* = 10), red clover (*n* = 9), or purple tansy (*n* = 10) plots per assessment period over all observation days (*n* = 7). Columns with the same letter are not significantly different at *α* = 0.05.

Over all observation days, significantly more workers were observed foraging on buckwheat than on red clover (*P* < 0.0001) and purple tansy (*P* = 0.0001) per assessment (Fig. 3A). Similarly, more workers entered or exited colonies per 10 min assessment period on buckwheat plots compared to colonies on red clover (*P* < 0.0001) or purple tansy plots (*P* < 0.0001; Fig. 3B). There was no difference between red clover and purple tansy plots in the number of active foragers (*P* = 0.9872; Fig. 3A) or the number of workers entering and exiting colonies (*P* = 0.9995; Fig. 3B).

### *Bombus impatiens* colony development

**Figure 4:**
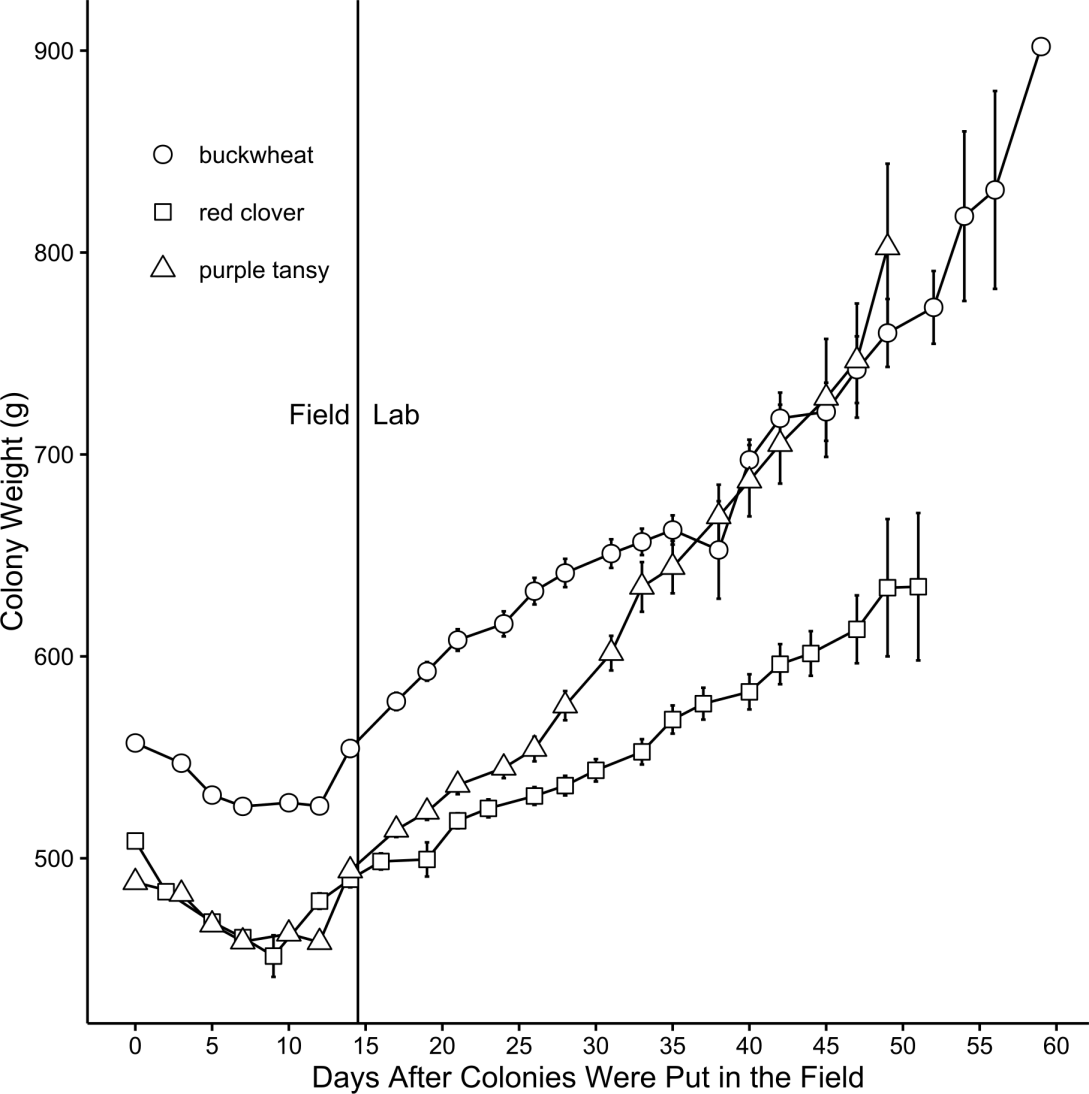
Mean weight (g) (± SE) of *Bombus impatiens* colonies by observation day (days on which colonies were weighed). Colonies were initially confined to flowering field plots of buckwheat (*n* = 10), red clover (*n* = 9), or purple tansy (*n* = 10) in the field. After 16 days in the field, which included four observation days, colonies were brought to the lab and maintained in a growth cabinet until two weeks after the first emergence of a new queen.

In the field, all colonies regardless of plant type tended to initially lose weight but then stabilize near the end of the field period. After transfer to the lab, colonies gained weight for the remainder of the study (Fig. 4). Although plant type had no effect on average colony weight during the field (*F* = 0.244; *df* = 2, 54; *P* = 0.784) or lab portion (*F* = 0.740; *df* = 2, 19; *P* = 0.492) of the study, colony weight at the start of the experiment did significantly affect average colony weight throughout the entire study (field: *F* = 12.14; *df* = 1, 55; *P* = 0.0009; lab: *F* = 388.85; *df* = 1, 23; *P* < 0.0001; Fig. 4). However, the plant type × starting weight interaction was not significant (field: *F* = 0.257; *df* = 2, 54; *P* = 0.7745; lab: *F* = 0.310; *df* = 2, 18; *P* = 0.7354). Observation day was significant for both the field (*F* = 215; *df* = 2, 21; *P* < 0.0001) and lab (*F* = 389; *df* = 2, 23; *P* < 0.0001) portions of the study, and there was a significant interaction between observation day and plant type (field: *F* = 14.4; *df* = 2, 21; *P* = 0.000161; lab: *F* = 18.5; *df* = 2, 23; *P* < 0.0001). During the field portion of the study, colonies on red clover plots lost weight more quickly on average than colonies on buckwheat or purple tansy plots, while during the lab portion of the study, colonies from purple tansy plots gained weight more quickly than those from buckwheat or red clover plots (Fig. 4).

**Table 1:** Range of total numbers of individuals per *Bombus impatiens* colony. Range of total numbers of immature stages (eggs, larvae, and pupae) and adult workers, males, and queens in *Bombus impatiens* colonies that were restricted to foraging on flowering buckwheat (*n* = 10), red clover (*n* = 7), or purple tansy (*n* = 10) for 16 days at the beginning of their colony cycle. Colonies were then maintained in a growth cabinet until two weeks after the first emergence of a new queen and then dissected.

Plant type	Range of total numbers of immature stages and adults per colony						
	Eggs	Larvae	Queen pupae	Male or worker pupae	Adult workers	Adult males	Adult queens
Buckwheat	0–49	41–171	0–21	6–54	13–138	1–80	2–28
Red clover	7–56	1–118	0–5	2–57	6–66	0–89	0–27
Purple tansy	12–48	10–189	0–15	1–68	59–189	0–174	0–21

**Table 2:** Mean number of *Bombus impatiens* individuals per colony. Mean (±SE) number of immature stages (eggs, larvae, and pupae) and adult workers, males, and queens in *Bombus impatiens* colonies that were restricted to foraging on flowering buckwheat (*n* = 10), red clover (*n* = 7), or purple tansy (*n* = 10) for 16 days at the beginning of their colony cycle. Colonies were then maintained in a growth cabinet until two weeks after the first emergence of a new queen and then dissected. Means within columns with the same letter are not significantly different at *α* = 0.05 (Tukey test).

Plant type	Mean (±SE) number of immature stages and adults per colony						
	Eggs	Larvae	Queen pupae	Male or worker pupae	Adult workers	Adult males	Adult queens
Buckwheat	29 ± 5.8a	93 ± 12.6a	3 ± 2.1a	36 ± 4.9a	56 ± 12.0b	28 ± 9.9a	9 ± 2.5a
Red clover	29 ± 7.1a	60 ± 18.1a	1 ± 0.72a	27 ± 8.0a	28 ± 5.0b	41 ± 13.1a	9 ± 4.1a
Purple tansy	32 ± 3.4a	98 ± 16.2a	4 ± 1.9a	35 ± 6.9a	108 ± 15.7a	33 ± 16.7a	11 ± 2.5a

**Table 3:** Mean weight of *Bombus impatiens* adults per colony. Mean (±SE) weight (g) of adult workers, males, and new queens from *Bombus impatiens* colonies that were restricted to foraging on flowering buckwheat (*n* = 10), red clover (*n* = 7), or purple tansy (*n* = 10) for 16 days at the beginning of their colony cycle. Colonies were then maintained in a growth cabinet until two weeks after the first emergence of a new queen and then dissected. Means within columns with the same letter are not significantly different at *α* = 0.05 (Tukey test).

Plant type	Mean (±SE) adult weight (g) per colony		
	Workers	Males	Queens
Buckwheat	0.15 ± 0.00533ab	0.18 ± 0.00959a	0.62 ± 0.0257a
Red clover	0.14 ± 0.00673b	0.17 ± 0.0109a	0.68 ± 0.0369a
Purple tansy	0.16 ± 0.00532a	0.16 ± 0.0102a	0.61 ± 0.0267a

Within each plant type, there was high variability in the number of immature and adult individuals per colony (Table 1), and colonies tended to skew their production of reproductives towards queens or males. Plant type had no effect on the number of immature stages or adults per colony with one exception: colonies from purple tansy plots contained significantly more adult workers compared with colonies from buckwheat (*w* = 82.5; *P* = 0.0155) or red clover (*w* = 70.0; *P* = 0.0001) plots (Table 2). Two colonies from purple tansy plots and one colony from a red clover plot did not produce new queens. Plant type had a significant effect on adult worker weight, with workers from colonies foraging on purple tansy weighing more than workers from red clover colonies (*P* = 0.0204; Table 3). In contrast, queen (*F* = 1.16; *df* = 2, 20; *P* = 0.333) and male (*F* = 0.847; *df* = 2, 19; *P* = 0.444) weight did not differ with plant type (Table 3).

## Discussion

Under our experimental conditions, *B. impatiens* foraging activity differed with plant type: both the number of active foragers and number of foragers entering or exiting colonies were at least 2× higher on buckwheat plots compared with colonies on purple tansy and red clover plots. This was surprising given that there were no differences between treatments in colony weight during the experiment, and that all three plants are known forage plants for bumble bees and other bees (Williams, 1997; Carreck & Williams, 2002; Pontin et al., 2006; Jacquemart, Gillet & Cawoy, 2007; Colla & Dumesh, 2010; Bartomeus et al., 2014). Indeed, we qualitatively observed during data collection that flowering buckwheat plants outside of our enclosures were continuously heavily visited not only by wild bumble bees (*Bombus* spp.), but also honey bees, eastern carpenter bees (*Xylocopa virginica* L.), and various species of solitary bees (Halictidae and Megachilidae). However, consistent with the low foraging rates we observed on our red clover plots, we observed far less wild bee or honey bee visitation, in terms of both numbers of individuals and species, to the flowering red clover plants outside of our enclosures. The specific reason(s) for these observations is unclear. It is possible that Japanese beetle feeding damage to the red clover flowers and/or the fact that the plants were flattened by the storm early in August contributed to lower bee visitation rates inside and outside of our plots. Interestingly, although foraging activity on purple tansy plots was low compared to buckwheat plots, wild bumble bees and honey bees were present on purple tansy flowers outside of the plots in numbers similar to those observed on buckwheat. This suggests some factor(s) specific to the colonies or plants within our purple tansy plots may have resulted in a comparatively lower foraging activity. We did not notice a difference in health, flower availability, or insect pest damage between the purple tansy plants inside and outside our plots; thus, it is unlikely that some characteristic of the plants on our purple tansy plots reduced foraging. Similarly, there was no indication colonies on purple tansy plots differed in terms of health or behaviour from the rest of the colonies used in our study.

All colonies from buckwheat and purple tansy plots survived for the duration of our study and appeared to develop normally. However, the foundress queen in three colonies from red clover plots died. The reason(s) for these queen deaths are unclear. Foraging activity was low on red clover plots, and therefore a lack of food resources may have contributed to the death of the first queen in the field. However, we did not observe a concurrent loss of workers or brood in that colony. Furthermore, the other two foundress queens died during the lab portion of our study when food resources were unlimited. Thus unidentified behavioural and/or physiological factors unrelated to their confinement to red clover plots likely contributed to the death of the three queens.

Among colonies with surviving foundress queens, development in terms of number of individuals per colony was similar on all three plant types. In particular, the number of queens produced per colony, a critical endpoint recommended for assessing the impacts of pesticides on bumble bee colonies (Cabrera et al., 2016), did not differ among plant types. However, two purple tansy colonies did not produce any new queens. These same two colonies also contained an abundance of workers (181 and 189) compared to all other colonies in our study, which inflated the mean number of workers per colony we observed for purple tansy (Table 1). Bumble bees display high inter-colony variability in the number of individuals from each caste produced per colony (Husband, 1977; Cnaani, Schmid-Hempel & Schmidt, 2002; Goulson et al., 2002), and correspondingly, the total number of individuals of each immature and adult stage per colony varied greatly within each plant type in our study. Therefore, the high number of workers and lack of queens produced in these two colonies may simply reflect this natural variability.

Corresponding to the number of individuals per colony, we did not observe a difference in colony weight due to plant type. Interestingly, all colonies, regardless of plant type, initially lost weight during the field portion of our study. During this time, we did not observe a concurrent loss of workers or brood, and thus weight loss did not seem to reflect a decline in colony health. However, colony weight loss generally corresponded with an increase in foraging activity (Figs. 1 and 3), and thus the loss partially can be attributed to foragers that were absent from the colony during weighing. Additional weight may have been lost as colonies consumed stored honey and pollen to compensate for a lack of incoming food resources, either initially, before foragers began visiting the plants (colonies on buckwheat plots) or continually for colonies that consistently displayed low foraging activity (colonies on red clover or purple tansy plots).

Despite exhibiting a lower foraging rate, purple tansy colonies gained weight more quickly while in the lab compared to buckwheat colonies. While purple tansy produces pollen that is of high nutritional quality for bees (Pernal & Currie, 2000), buckwheat pollen is of lower quality (Sommerville, 2001). Thus, it is possible that because they had access to a higher quality resource early in their development, purple tansy colonies were able to gain weight faster. Yet, red clover pollen also is high quality (Hanley et al., 2008; Somme et al., 2015), and red clover colonies gained weight more slowly despite exhibiting a foraging rate similar to purple tansy colonies. Therefore, resource quality during the field portion of our study likely cannot solely account for the comparatively fast weight gain and development of purple tansy colonies, and further study is required to determine if the rapid colony weight gain we observed is truly related to foraging from purple tansy or simply a coincidence.

Our results suggest that buckwheat, red clover, and purple tansy may not be equally appropriate as surrogate plants in semi-field studies using small enclosures with *B. impatiens*. During semi-field pesticide risk assessments, the surrogate plant must elicit high foraging activity to ensure that colonies are exposed to the test product, and we observed consistently higher foraging on buckwheat plots. To detect any detrimental effects of the test product, the plants must produce an adequate supply of quality nectar and pollen to allow for optimal colony development during the test period. Other than a higher number of workers in purple tansy colonies, we did not observe a statistical difference in colony development in terms of numbers of individuals per colony and colony weight, which suggests that all three plant types can sustain *B. impatiens* colonies under semi-field conditions. However, colonies from red clover plots generally contained fewer individuals and developed more slowly. In contrast, colonies from buckwheat and purple tansy plots produced an abundance of brood and adults, and all buckwheat colonies produced new queens. Thus, in terms of foraging activity and colony development, buckwheat was preferable to red clover and purple tansy in our study. In our experience, buckwheat also was more favourable in terms of growth characteristics, cost, and maintenance. Buckwheat grew rapidly and flowered longest. It also was the least expensive plant overall in terms of seed and maintenance costs. Finally, unlike red clover and purple tansy, buckwheat did not experience weed pressure or insect pest damage (although we recognize that pest pressure on any of the three plant types will vary with geographic region and environmental conditions). Therefore, to ensure forager pesticide exposure, adequate colony development, and favourable plant growth, we suggest buckwheat as a surrogate plant for use in semi-field pesticide toxicity assessments with *B. impatiens*, and possibly other bumble bees.

##  Supplemental Information

10.7717/peerj.2228/supp-1 Supplemental Information 1 Planting details Seeding details, date of first flower, and dates that *Bombus impatiens* colonies were introduced or removed from plots of each plant type (buckwheat, red clover, and purple tansy).

10.7717/peerj.2228/supp-2 Data S1 Raw data on *Bombus impatiens* foraging activity and colony development Raw data on *Bombus impatiens* foraging activity and colony development (numbers of individuals per colony and colony weight) on small plots of flowering buckwheat, red clover, and purple tansy.

## Additional Information and Declarations

### Data Availability

The following information was supplied regarding data availability: The raw data have been supplied as Data S1 .
